# Mitigation of acrylamide in cookies and crispbread using calcium salts and phenolic acids in combination with asparaginase as well as rosemary extract^☆^

**DOI:** 10.1016/j.fochx.2025.102605

**Published:** 2025-05-30

**Authors:** Shpresa Musa, Claudia Oellig, Katharina Anne Scherf

**Affiliations:** a Department of Bioactive and Functional Food Chemistry, Institute of Applied Biosciences, Karlsruhe Institute of Technology (KIT), 76131, Karlsruhe, Germany; b Department of Food Chemistry and Analytical Chemistry, Institute of Food Chemistry, University of Hohenheim, 70599, Stuttgart, Germany; c Leibniz Institute for Food Systems Biology at the Technical University of Munich, 85354, Freising, Germany; d Technical University of Munich, TUM School of Life Sciences, Professorship of Food Biopolymer Systems, 85354, Freising, Germany

**Keywords:** Color, Cereal-based products, Ellagic acid, Gallic acid, Sensory analysis, Texture

## Abstract

Acrylamide is formed during high-temperature treatment in foods and presents a significant health and regulatory challenge. This study evaluates the effects of calcium salts and phenolic acids alone or in combination with asparaginase and rosemary extract in wheat cookies and rye crispbread. Acrylamide content, product color, texture, and sensory properties were assessed. When calcium salts and phenolic acids were used alone, acrylamide was reduced by 46 % and 50 % compared to the control. A combination of these with asparaginase resulted in a reduction of acrylamide by up to 89 % using ellagic acid. Specific treatments reduced cookie hardness, but asparaginase addition reversed this effect. Color mainly remained unaffected. Sensory analysis of selected treatments confirmed no significant changes in cookie aroma, taste, color, texture, and acceptability. This work provides a new approach by combining selected treatments to mitigate acrylamide while preserving product quality.

## Highlights

• Acrylamide reduction was achieved with calcium salts and phenolic acids.
• A combination of these with asparaginases was even more effective.
• Minimal changes in the color of cookies and crispbread were observed.
• Texture effects were improved with asparaginase addition.
• There were no changes in the sensory properties of cookies.

## 1 Introduction

Acrylamide is formed during high-temperature food preparation, particularly in carbohydrate-rich foods via the Maillard reaction between free asparagine and reducing sugars (Granvogl & Schieberle, 2006; Mottram, Wedzicha, & Dodson, 2002; Stadler et al., 2002; Tareke, Rydberg, Karlsson, Eriksson, & Törnqvist, 2002). Acrylamide was first detected in baked and fried foods in 2002 by researchers in Sweden (Swedish National Food Administration, 2002; Tareke et al., 2002). This finding sparked a significant health concern due to the 2 A classification of acrylamide as probably carcinogenic to humans (IARC, 1994). The European Food Safety Authority (EFSA) found that the daily exposure in the European population of adolescents, adults, the elderly, and the very elderly amounts to 0.4 to 0.9 μg/kg (EFSA, 2015). This is why Commission Regulation 2017/2158 of the European Union established mitigation measures and benchmark levels for various foods (European Union, 2017). Based on this Regulation, a benchmark of 350 μg/kg of acrylamide applies to cookies and crispbread.

Numerous studies have focused on investigating the acrylamide-forming potential in different baked goods (Amrein, Schönbächler, Escher, & Amado, 2004; Fredriksson, Tallving, Rosén, & Åman, 2004; Granvogl & Schieberle, 2006; Hendriksen, Kornbrust, Østergaard, & Stringer, 2009; Jung, Choi, & Ju, 2003; Mestdagh, de Wilde, Delporte, van Carlos, & de Meulenaer, 2008). The three main approaches for acrylamide reduction focus on agronomy, the recipe, and processing conditions (FoodDrinkEurope, 2019). The content of free asparagine in the raw material, such as cereal grains, is the crucial factor in agronomy determining the acrylamide content in the final product. Breeding efforts to control asparagine show promise because the content of asparagine varies greatly depending on species, cultivars, and growing conditions. The use of raw materials with a low asparagine content has been a focus of a few studies (Oddy et al., 2023; Raffan & Halford, 2019; Swiacka et al., 2024). Recipe-related strategies mostly center on the ingredients, such as using white flour instead of wholemeal flour due to the higher acrylamide-forming potential in wholemeal flour associated with the higher free asparagine content in wheat bran, switching to sodium bicarbonate as a raising agent rather than ammonium bicarbonate, altering the type of sugar, such as replacing fructose with glucose or non-reducing sugars, and including asparaginase in the recipe. Among the processing techniques are low heat input, fermentation, and novel heat treatment techniques (FoodDrinkEurope, 2019). One of the most common approaches is to reduce thermal input, which has been proven to be an effective way of mitigating acrylamide in cereal-based products (Bråthen & Knutsen, 2005; Mesias, Delgado-Andrade, & Morales, 2024; Mustafa, Andersson, Rosén, Kamal-Eldin, & Åman, 2005; Perestrelo et al., 2024; Wu, Yan, Cao, & Yuan, 2024).

Bakery products are responsible for the highest dietary acrylamide intake (EFSA, 2015). Therefore, many studies have investigated different strategies for reducing acrylamide in cereal-based products. Acrylamide mitigation can be achieved by adapting the recipe, including pH decrease and fermentation, and adding ingredients such as organic acids, calcium salts, and co-ingredients. Some studies showed that the addition of calcium salts could have a positive impact on mitigating acrylamide (Açar, Pollio, Di Monaco, Fogliano, & Gökmen, 2012; Batuwita, Jayasinghe, Marapana, & Jayasinghe, 2024; Gökmen, Açar, Köksel, & Acar, 2007; Kukurová, Ciesarová, Bednáriková, & Marková, 2009; Liang, Wang, & Sung, 2014) with a reduction of up to 60 % in model cookies. Another focus has been on adding polyphenols and plant extracts, representing a promising strategy for mitigating acrylamide (Mildner-Szkudlarz, Różańska, Piechowska, Waśkiewicz, & Zawirska-Wojtasiak, 2019; Oral, Dogan, & Sarioglu, 2014). Furthermore, the addition of asparaginases (Anese, Quarta, & Frias, 2011; Hendriksen et al., 2009; Kukurová, Morales, Bednáriková, & Ciesarová, 2009; Masciola et al., 2025; Musa, Becker, Oellig and Scherf, 2024a, Musa, Becker, Oellig and Scherf, 2024b; de Souza, Chávez, Fioravante Guerra, Gottschalk, & Freitas-Silva, 2025), replacement of reducing sugars (Amrein et al., 2004), and ammonium salts (Amrein, Andres, Manzardo, & Amado, 2006) has been investigated. However, combining these strategies with asparaginase remains unexplored.

We hypothesized that combining salts and phenolic acids with asparaginase would reduce acrylamide more effectively than asparaginase alone. To test this, the study aimed to evaluate the effectiveness of calcium chloride, calcium carbonate, ellagic acid, and gallic acid, individually and in combination with asparaginase, in mitigating acrylamide formation in wholemeal wheat cookies and wholemeal rye crispbread. Additionally, the potential of incorporating rosemary extract was explored. The effects of these treatments on color and texture were assessed, along with any sensory alterations in cookies. To our knowledge, our study is the first to systematically evaluate the combined effects of these mitigation strategies in cereal-based products, specifically wholemeal wheat cookies and wholemeal rye crispbread. While Regulation (EC) No 1333/2008 lists only calcium carbonate as an additive for cereal-based foods, we further investigated the efficacy of calcium chloride and phenolic acids in reducing acrylamide. These compounds have shown promising results in other food matrices and are either approved for different food applications or recognized for their antioxidant properties. By generating robust scientific data, we aim to provide insights that could support their potential inclusion in future regulatory frameworks, offering additional tools for the industry to enhance food safety and quality (European Commission, 2008).

## 2 Materials and methods

### 2.1 Reagents and ingredients

All chemicals and reagents were of analytical grade or higher. Baking powder (consisting of E 450 (diphosphates), E 500 (sodium carbonates), and starch), sugar, salt, and sunflower oil were bought from a local supermarket. Wholemeal wheat and rye flour were provided by IREKS (Kulmbach, Germany). Calcium chloride (CaCl_2_), calcium carbonate (CaCO_3_), gallic acid (GA), and ellagic acid (EA) were purchased from Thermo Fisher Scientific (Waltham, MA, USA) or VWR (West Chester, PA, USA). Rosemary extract (RE) was purchased from MIVALIED (Buchholz, Germany). Asparaginase Acrylaway L was donated by Novonesis (Bagsværd, Denmark). The Acrylamide ES ELISA kit and Acrylamide derivatization kit were acquired from Eurofins (Eurofins Scientific SE, Luxembourg), and ISOLUTE Multimode (500 mg/3 mL) and ISOLUTE ENV+ (200 mg/3 mL) were from Biotage (Uppsala, Sweden).

### 2.2 Addition levels of salts, phenolic acids, rosemary extract, and asparaginases

For cookies, CaCl_2,_ CaCO_3,_ ellagic acid, and gallic acid were added to the dough at three amounts: 100 μmol, 50 μmol, and 5 μmol, respectively. Each ingredient was added alone or in combination with 50 mg/kg Acrylaway L (abbreviated as R). For crispbread, only CaCl_2_ and ellagic acid were tested using a combination of 100 μmol, 50 μmol, and 5 μmol, with R. CaCl_2_ was selected due to its better solubility in water compared to CaCO_3_. Ellagic acid was chosen due to better acrylamide reductions than gallic acid in cookies. In cookies and crispbread, rosemary extract was tested without adding asparaginase using three amounts: 0.01 %, 0.1 %, and 0.2 %. The lower levels were selected due to the possible altered sensory properties in baked goods and color changes. For an overview of all samples tested and specific amounts used, please refer to Table 1, Table 2 for cookies and crispbread, respectively.

**Table 1:** Color analysis of wheat cookies using CCell. The color is represented in terms of L* (lightness), a* (green-red), b* (blue-yellow) values and the color difference (ΔE) between control and treatment.

Treatment	Amount	L*-value	a*-value	b*-value	ΔE
Control (C-10)^1^	NA	20.6 ± 4.3	8.5 ± 1.1	18.2 ± 2.0	0
CaCl_2_	100 μmol	23.5 ± 4.2	9.2 ± 0.8	19.0 ± 1.6	5.2
	50 μmol	26.1 ± 3.1^#^	9.1 ± 1.0	19.4 ± 1.3	5.7
	5 μmol	25.9 ± 2.2^#^	9.2 ± 1.3	19.7 ± 2.0	5.5
	100 μmol + R	23.5 ± 3.4	9.2 ± 1.2	19.1 ± 1.4	3.8
	50 μmol + R	23.3 ± 5.6	9.0 ± 1.1	19.3 ± 1.9	4.7
	5 μmol + R	23.3 ± 2.4	9.8 ± 0.3^#^	19.7 ± 1.5	4.4
CaCO_3_	100 μmol	22.7 ± 5.5	7.2 ± 0.5	17.7 ± 2.3	4.1
	50 μmol	22.1 ± 7.6	7.4 ± 1.8	16.8 ± 3.4	2.7
	5 μmol	24.8 ± 4.1	8.6 ± 1.0	19.0 ± 1.3	2.5
	100 μmol + R	23.5 ± 2.0	9.0 ± 0.9	18.9 ± 0.8	6.3
	50 μmol + R	22.9 ± 3.4	8.5 ± 0.6	17.8 ± 0.8	3.9
	5 μmol + R	24.4 ± 2.6	9.4 ± 0.9	19.3 ± 1.2	5.2
Gallic acid	100 μmol	26.9 ± 2.8^#^	7.6 ± 1.5	17.5 ± 2.4	1.3
	50 μmol	24.4 ± 1.9	8.8 ± 1.0	19.1 ± 1.4	1.4
	5 μmol	25.6 ± 4.9^#^	7.6 ± 1.2	16.9 ± 2.2	4.3
	100 μmol + R	20.6 ± 3.4	9.2 ± 1.3	19.2 ± 1.7	3.1
	50 μmol + R	20.3 ± 4.1	9.1 ± 1.4	19.3 ± 1.9	5.6
	5 μmol + R	24.4 ± 4.2	7.3 ± 1.5	16.5 ± 2.5	5.6
Ellagic acid	100 μmol	24.4 ± 2.1	9.0 ± 1.5	18.7 ± 1.9	3.1
	50 μmol	25.3 ± 3.6^#^	8.8 ± 1.0	17.5 ± 0.9	2.9
	5 μmol	24.9 ± 2.5	9.3 ± 1.1	18.8 ± 2.0	3.3
	100 μmol + R	24.6 ± 1.7	8.8 ± 0.9	17.1 ± 0.8	2.5
	50 μmol + R	23.1 ± 2.8	9.3 ± 0.7	17.6 ± 1.4	2.2
	5 μmol + R	22.7 ± 4.9	8.5 ± 1.4	16.9 ± 1.6	4.3
Rosemary extract	Control (C-RE)^2^	20.3 ± 4.8	8.7 ± 1.4	18.3 ± 1.8	0
	0.01 %	25.3 ± 2.0^#^	9.2 ± 0.8	19.9 ± 0.8	3.0
	0.1 %	26.0 ± 2.4^#^	8.4 ± 0.9	19.0 ± 1.4	2.3
	0.2 %	25.9 ± 5.4^#^	8.6 ± 1.7	18.6 ± 2.6	4.1

The superscript # indicates a significant difference compared to the control (ANOVA with Dunnett's t-test p ≤ 0.05, n = 6).

NA, no addition; R, 50 mg/kg of asparaginase Acrylaway L.

1 Control sample incubated at 60 °C for 10 min for treatments including calcium chloride, calcium carbonate, gallic acid, and ellagic acid.

2 Control sample without incubation for rosemary extract treatment.

**Table 2:** Color analysis of rye crispbread using CCell. The color is represented in terms of L* (lightness), a* (green-red), b* (blue-yellow) values and the color difference (ΔE) between control and treatment.

Treatment	Amount	L*-value	a*-value	b*-value	ΔE
Control (C)	NA	29.9 ± 1.5	3.3 ± 0.6	7.9 ± 2.3	0
CaCl_2_	100 μmol + R	30.1 ± 1.7	3.0 ± 0.8	9.2 ± 2.6	3.9
	50 μmol + R	30.3 ± 0.8	3.9 ± 0.9	11.5 ± 2.8	2.2
	5 μmol + R	30.2 ± 1.8	2.6 ± 0.6	8.0 ± 1.9	4.0
Ellagic acid	100 μmol + R	30.3 ± 1.2	3.3 ± 0.4	11.3 ± 1.6	3.4
	50 μmol + R	30.3 ± 2.3	3.7 ± 1.1	9.6 ± 2.7	1.8
	5 μmol + R	30.8 ± 1.3	4.4 ± 1.5	11.0 ± 2.9	3.4
Rosemary extract	0.01 %	29.9 ± 1.4	3.8 ± 1.7	11.8 ± 3.0	1.3
	0.1 %	29.9 ± 1.7	3.4 ± 0.5	10.1 ± 1.7	3.7
	0.2 %	31.0 ± 1.4	4.9 ± 1.5	11.4 ± 3.8	0.8

There were no significant differences compared to the respective control (ANOVA with Dunnett's t-test p ≤ 0.05, n = 6).

NA, no addition; R, 50 mg/kg of asparaginase Acrylaway L.

### 2.3 Preparation of cookies and crispbread

Cookies were made using wholemeal wheat flour using a recipe reported earlier (Musa et al., 2024a). The dry components, flour, sugar, and baking powder in amounts of 100 g, 15.3 g, and 0.33 g, respectively, were mixed for 1 min at low speed using a food processor (Robert Bosch GmbH, Stuttgart, Germany). Water (48 mL) and sunflower oil (12.6 mL) were mixed with the dry components for 3 min at medium speed and 30 s using high speed. The dough was rolled to a thickness of 0.5 cm, and cookies with a diameter of 6 cm were cut out. Crispbread was prepared using wholemeal rye flour. For 100 g of flour, 77 mL of water and 1.15 g of salt were added. Crispbread dough was mixed at low speed for 30 s, medium speed for 60 s, and high speed for another 60 s. Then, the dough was rolled to a thickness of 0.5 cm and cut into rectangles with a length of 8 cm and a width of 4 cm. When treatments were applied, the amounts of CaCl_2_, CaCO_3_, ellagic acid, gallic acid, and rosemary extract mentioned under 2.2 were added directly during dough mixing. Cookies and crispbread samples that included a combination of treatment with asparaginase R were incubated at 60 °C for 10 min. Cookies were baked in the oven (Rossella XFT197, Unox S.p.A., Cadoneghe, Italy) at 220 °C for 11 min, and crispbread was baked at 240 °C for 13 min. Wheat flour is typically used for cookies, while rye flour is traditionally preferred for crispbread. Therefore, these flours were selected accordingly for each product.

### 2.4 Acrylamide analysis

An Acrylamide ES ELISA kit was used to quantify acrylamide in cookies and crispbread. The method was performed according to Musa, Becker, Oellig and Scherf, 2024a, Musa, Becker, Oellig and Scherf, 2024b and the manufacturer's test method provided in the kit (Gold Standard Diagnostics, 2021). Two grams of the sample were mixed well with 40 mL of distilled water for 30 min. The samples were left to rest for 5 min to allow sample sedimentation after mixing. Then, the sample was filtered and centrifuged for 5 min at 13,000 x g. Using Multimode SPE and ENV+ SPE columns, the sample underwent a clean-up procedure. Finally, acrylamide was eluted using a mixture of methanol/water (60:40 *v*/v). The eluted sample was then derivatized, and the ELISA was performed using a 96-well plate. The samples were placed in a photometer (Tecan, i-control infinite 200Pro, Männedorf, Switzerland) and measured at an absorbance of 450 nm.

### 2.5 Color analysis

To evaluate the color properties of baked products, the values L* (lightness), a* (green-red), and b* (blue-yellow), respectively, were obtained using CCell. The device was calibrated using a calibration card (CC006, Calibre Control, Warrington, UK) with two sides, a gray surface (neutral hue N8.5) and a ramp surface. After calibration, each sample was placed on a blue background for color measurement. A total of six measurements from three batches of each treatment were performed for each cookie and crispbread, respectively. Finally, the color difference between control and treatment (ΔE) was calculated according to Palazoğlu, Coşkun, Kocadağlı, and Gökmen (2012) using the following formula:

$$\Delta E=\sqrt{{\left(L^{*}-L_{0}^{*}\right)}^{2}+{\left(a^{*}-a_{0}^{*}\right)}^{2}+{\left(b^{*}-b_{0}^{*}\right)}^{2}}$$

For each value, 0 represents the color parameter of the control sample.

### 2.6 Texture properties

The assessment of hardness (N) and fracturability (mm) was done using a TA.XTplus texture analyzer (Stable Micro Systems, Godalming, UK) equipped with a 5 kg load cell (Musa et al., 2024a). We used a three-point bending rig (HDP/3 PB) and a heavy-duty platform (HDP/90) to break cookies and crispbread and assess textural properties. A method called “Measurement of the hardness and resistance of biscuits/cookies to bend or snap” was used, available in the Exponent software (Stable Micro Systems, Version 6.1.20.0, Godalming, UK) of the texture analyzer. A total of six measurements from three batches were taken for cookies and crispbread.

### 2.7 Sensory analysis

To evaluate any potential changes in the sensory properties of cookies, three treatments were selected and assessed: calcium chloride (CaCl_2_ 100 μmol + R), ellagic acid 100 μmol + R (EA 100 μmol + R), and rosemary extract (RE 0.1 %), respectively. A control sample was included as well. Thirteen untrained panelists were asked to evaluate color, texture (crunchiness, chewiness, and hardness), taste, aroma, and overall acceptability. The participants assessed each sensory attribute using a hedonic scale of 1 to 5 for each sample. They compared it to the reference, using the following range of descriptions: very dark (1) to very light (5) for color, not crunchy (1) to very crunchy (5) for crunchiness, very chewy (1) to not chewy (5) for chewiness, very hard (1) to very soft (5) for hardness, unpleasant (1) to very pleasant (5) for taste, unpleasant (1) to very pleasant (5) for aroma and dislike (1) to like very much (5) for overall acceptability. Finally, participants could add notes in the comment section for any off-taste or other product characteristics not included in the survey. All participants gave informed consent to participate in the sensory study and to use their information.

### 2.8 Statistical analysis

Microsoft Excel was used to calculate means and standard deviations. Origin 2023 (OriginLab Corporation, Northampton, MA, USA) and IBM SPSS Statistics (IBM Corp, Version 29.0, Armonk, NY) were used for statistical analysis. Dunnett's *t*-test (*p* ≤ 0.05) was utilized to identify significant differences between the control and treated samples. Sensory samples were analyzed using the non-parametric Kruskal-Wallis test (*p* < 0.05). Dunn's test was a post hoc method for pairwise comparisons with control and multiple treatment groups. Three technological replicates (i.e., batches) were prepared for each treatment. An online tool by the Arigo website was used to calculate the acrylamide content for each sample (ELISA calculator) (Arigo Biolaboratories, n.d).

## 3 Results

### 3.1 Acrylamide content in cookies

**Fig. 1:**
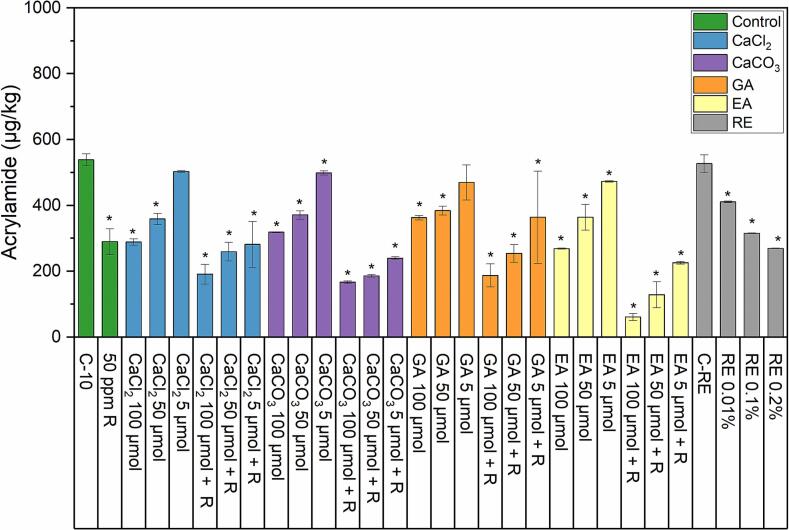
Acrylamide content of wholemeal wheat cookies with the addition of calcium chloride, calcium carbonate, gallic acid, ellagic acid, and rosemary extract. Error bars indicate the standard deviation of the mean, and asterisks indicate a significant difference compared to the respective control (ANOVA with Dunnett's *t-*test *p* ≤ 0.05, *n* = 3). Abbreviations as follows: CaCl_2_ (calcium chloride), CaCO_3_ (calcium carbonate), GA (gallic acid), EA (ellagic acid), RE (rosemary extract), R (50 mg/kg of asparaginase Acrylaway L).

To evaluate the impact of salts, phenolic acids, and rosemary extract on the reduction of acrylamide content in cookies, each of these was added using three different amounts (Table 1). Additionally, phenolic acids and salts were tested with asparaginase R (Fig. 1), for CaCl_2_ and CaCO_3_, a significant (p ≤ 0.05) reduction of acrylamide was observed compared to the control (C-10, 538 μg/kg of acrylamide) when these salts were added to cookies without R, except for 5 μmol of CaCl_2_. However, although the reduction was significant for the amount of 5 μmol of CaCO_3_, this was a relatively low reduction to 498 μg/kg of acrylamide. Overall, increasing the amounts of calcium salts to 100 μmol reduced acrylamide to 288 μg/kg (CaCl_2_) and 318 μg/kg (CaCO_3_). A combination of each salt with asparaginase R further improved the reduction because even the 5 μmol amounts of CaCl_2_ or CaCO_3_ + 50 mg/kg R showed a significant reduction of acrylamide to 281 μg/kg or 239 μg/kg, respectively. However, compared to 50 mg/kg R alone, this reduction was primarily associated with the asparaginase R rather than the salt. Ellagic acid and gallic acid were also tested alone or in combination with asparaginase R. Gallic acid showed lower reductions of acrylamide compared to ellagic acid. As with the salts, increasing the amount of either acid from 5 μmol to 100 μmol showed an improvement in acrylamide reduction. Ellagic acid reduced acrylamide to 268 μg/kg and gallic acid to 362 μg/kg at 100 μmol, to 364 μg/kg and 384 μg/kg at 50 μmol, and 472 μg/kg and 469 μg/kg at 5 μmol, for ellagic acid and gallic acid, respectively. The addition of asparaginase R in combination with gallic acid or ellagic acid further reduced acrylamide, with the highest reduction to 61 μg/kg achieved by the treatment with ellagic acid (EA 100 μmol +50 mg/kg R). Rosemary extract was also tested in cookies using three amounts: 0.01 %, 0.1 %, and 0.2 %, respectively. All amounts achieved significant reductions compared to the control (C-RE, 526 μg/kg), with the highest reduction to 269 μg/kg of acrylamide achieved by adding 0.2 % of rosemary extract. Comparing the control for phenolic acids and calcium salts incubated for 10 min (C-10) with the rosemary extract control (C-RE), which was not incubated before baking, reveals a negligible difference in acrylamide levels. The acrylamide content was 538 μg/kg in C-10 and 526 μg/kg in C-RE. This indicates that incubating the dough at 60 °C for 10 min does not significantly influence acrylamide formation. These findings demonstrate the excellent reproducibility of the baking trials and further validate the reliability of the entire process.

### 3.2 Acrylamide content in crispbread

**Fig. 2:**
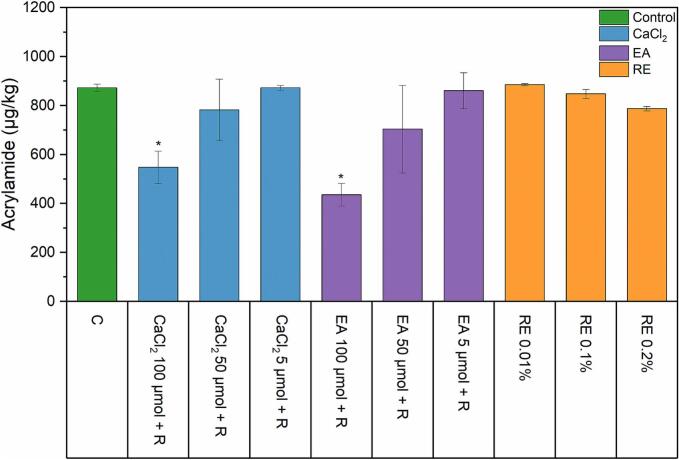
Acrylamide content of wholemeal rye crispbread with the addition of calcium chloride, ellagic acid, and rosemary extract. Asterisks indicate a significant difference compared to the control (ANOVA with Dunnett's *t-*test p ≤ 0.05, *n* = 2). Abbreviations as follows: CaCl_2_ (calcium chloride), EA (ellagic acid), RE (rosemary extract), R (50 mg/kg of asparaginase Acrylaway L).

In crispbread, only two treatments resulted in a significant reduction of acrylamide (Fig. 2). The combination of 100 μmol CaCl_2_ + 50 mg/kg R showed a significant reduction of acrylamide to 547 μg/kg, while 50 μmol or 5 μmol of CaCl_2_ with R showed minimal or no reduction at all compared to the control with 872 μg/kg. Like CaCl_2,_ ellagic acid caused a significant decrease of acrylamide to 435 μg/kg only for the combination of 100 μmol +50 mg/kg R, and minimal or no reduction at all using 50 μmol and 5 μmol, respectively. Rosemary extract showed a minimal decrease at the amount of 0.2 %, but this was statistically insignificant.

### 3.3 Evaluation of color in cookies and crispbread

To evaluate any effect of the treatments on the color properties of cookies and crispbread, the L*-, a*-, and b*-values of each treatment were compared to the respective control using ANOVA with Dunnett's *t-*test (*p* ≤ 0.05) (Table 1, Table 2). The addition of rosemary extract caused a significant increase in the L*-value, but there were no significant changes in a*- or b*-values. The application of ellagic acid resulted in minor color differences for the cookies. Only 50 μmol of ellagic acid showed an increase in L*-value, which was small but statistically significant. None of the other treatments with ellagic acid affected the L*-, a*-, or b*-value. Gallic acid also had a minimal effect on the color of cookies. Only the addition of 100 μmol or 5 μmol of gallic acid showed a significant increase in L*-value. CaCl_2_ showed a slightly larger impact on the color properties of cookies than CaCO_3_; however, combining any of the treatments with asparaginase R did not impact any color properties.

Table 2 presents crispbread's L*-, a*-, and b*-values. Different from cookies, the addition of rosemary extract showed no significant differences in color compared to the control. Adding ellagic acid and CaCl_2_ showed no significant differences in L*-, a*-, or b*-values.

### 3.4 Textural properties of cookies and crispbread

**Fig. 3:**
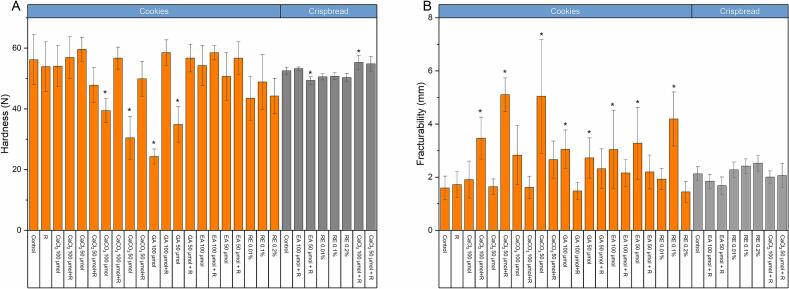
Hardness (N) and fracturability (mm) of wholemeal wheat cookies and rye crispbread with the addition of calcium chloride, calcium carbonate, gallic acid, ellagic acid, and rosemary extract. Error bars indicate the standard deviation of the mean, and asterisks indicate a significant difference compared to the respective control (ANOVA with Dunnett's *t*-test p ≤ 0.05, *n* = 6). Abbreviations as follows: CaCl_2_ (calcium chloride), CaCO_3_ (calcium carbonate), GA (gallic acid), EA (ellagic acid), RE (rosemary extract), R (50 mg/kg of asparaginase Acrylaway L).

The evaluation of textural properties, including hardness and fracturability, was done for all treatments applied to cookies and crispbread. Tables S1 and S2 include the 5 μmol treatment; however, this treatment was excluded from Fig. 3, as the treatment had minimal impact on the texture properties. Overall, most treatments showed minimal cookie changes, which were statistically insignificant. However, four treatments showed a significant decrease in hardness (Fig. 3A): CaCO_3_ at 100 μmol and 50 μmol, and gallic acid at 100 μmol and 50 μmol. However, when these treatments were combined with asparagine R, the hardness showed no significant change compared to the control. Ellagic acid and rosemary extract caused minimal changes in the hardness of cookies, none of which were statistically significant.

Crispbread showed minimal changes in texture compared to the control due to the treatments applied (Fig. 3A). Only adding CaCl_2_ 100 μmol + R and EA 50 μmol + R resulted in a significant increase and decrease, respectively. Rosemary extract caused no significant changes in hardness or fracturability.

The fracturability of cookies and crispbread is presented in Fig. 3B. Some treatments showed a significant increase in the fracturability of cookies in comparison to the control. The most impactful treatments regarding the fracturability of cookies were CaCl_2_ at 50 μmol + R, CaCO_3_ at 50 μmol, and RE 0.1 %, respectively. A high variability between the three batches was found, as shown in the error bars in Fig. 3B. In crispbread, there were only minimal changes in fracturability, none of which were statistically significant.

### 3.5 Sensory analysis

**Fig. 4:**
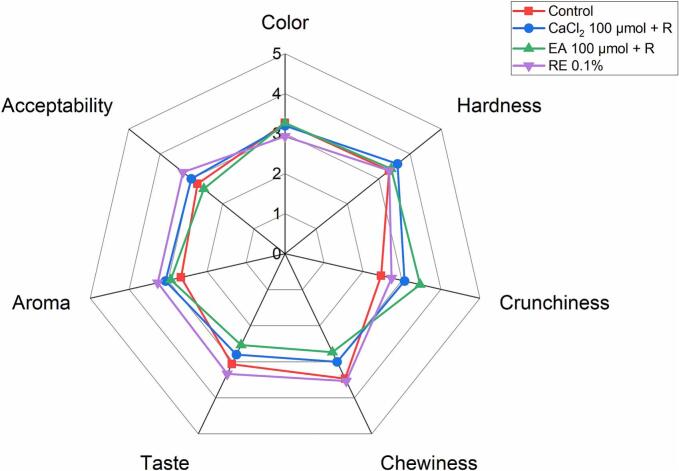
Sensory evaluation including color, texture, taste, aroma, and overall acceptability for wholemeal wheat cookies. Results are presented as the mean scores of 13 panelists. Samples were analyzed using the non-parametric Kruskal-Wallis test (*p* < 0.05) and Dunn's test was used as a post hoc method for pairwise comparisons with control and multiple treatment groups. Abbreviations as follows: CaCl_2_ (calcium chloride), EA (ellagic acid), RE (rosemary extract), R (50 mg/kg of asparaginase Acrylaway L).

A sensory evaluation was performed with 13 participants to assess the impact of the treatments applied to baked products. Cookies were selected for the sensory assessment due to their more pronounced sensory attributes, providing a more suitable medium to assess the effects of the treatments. Crispbread, being more neutral in flavor and texture, may not have shown a clear difference, thus limiting the effectiveness of the evaluation in detecting treatment impacts. Three treatments were selected: rosemary extract 0.1 %, ellagic acid 100 μmol + R, and CaCl_2_ 100 μmol + R, respectively, and compared to the control sample. The ellagic acid and CaCl_2_ treatments (100 μmol + R) were selected because they provided the highest acrylamide reduction, compared to 50 μmol or 5 μmol + R. While rosemary extract at 0.1 % reduced acrylamide slightly less compared to 0.2 %, higher amounts of rosemary extract were avoided as they were assumed to have a more significant impact on the taste of the cookies. This selection aimed to balance acrylamide reduction with the preservation of product quality. The sensory attributes color, hardness, crunchiness, chewiness, taste, aroma, and overall acceptability were evaluated and presented in Fig. 4. Overall, none of the treatments showed any significant changes in sensory attributes compared to the control. The participants also observed that cookies with 0.1 % rosemary extract were slightly darker than the control sample. Ellagic acid showed a slight increase in crunchiness compared to the control sample. CaCl_2,_ in general, showed improved taste, aroma, and overall acceptability compared to other samples. The participants also noted this in the comment section. Although these slight changes are visible in the spiderweb graph (Fig. 4), statistically, none were significant compared to the control.

## 4 Discussion

Acrylamide reduction by applying various compounds has reduced or minimized acrylamide in different products (Açar et al., 2012; Miśkiewicz, Nebesny, Rosicka-Kaczmarek, Żyżelewicz, & Budryn, 2018; Narita & Inouye, 2014). While several compounds can be added to lower acrylamide levels in baked products, one of the current study's strategies was using calcium salts because they are often used in flour fortification (*BAKERpedia*, 2021). Calcium salts enhance the nutritional profile of flour by contributing to the dietary calcium intake of populations consuming baked goods made of fortified flour. Besides, the cations prevent the formation of the Schiff base of asparagine during the Maillard reaction, which is the main intermediate that forms acrylamide in the final product (Gökmen & Şenyuva, 2007). Adding CaCl_2_ reduced acrylamide by up to 46 % (CaCl_2_ 100 μmol) in cookies. Another study that investigated the ability of CaCl_2_ to reduce acrylamide in cereal model systems showed that a reduction of up to 90 % is possible (Kukurová, Ciesarová, et al., 2009). In the current study, increasing levels of calcium salts in cookies further decreased acrylamide, considering the three levels tested. However, amounts beyond 100 μmol were not evaluated. Future research should explore higher amounts (e.g., 200 μmol) to investigate whether acrylamide reduction continues. It is also important to consider potential sensory implications, as high levels of calcium salts may introduce off-tastes to the product. Therefore, sensory analysis at higher calcium salt amounts is essential to ensure product acceptability. In agreement with our study, Açar et al. (2012) also reported a decrease in acrylamide when the amount of CaCl₂ was increased. CaCl₂ also showed a significant reduction of acrylamide in potato products, with a reduction of 95 % in fried potato strips (Gökmen & Şenyuva, 2007).

In the current study, CaCO₃ achieved a slightly lower acrylamide reduction compared to CaCl₂. At an amount of 100 μmol, CaCO₃ reduced acrylamide by up to 41 %, whereas CaCl₂ achieved a reduction of 46 % compared to the control sample. Increasing amounts of CaCO₃ also showed a significant decrease in acrylamide in white flatbread (Alsharafani et al., 2023). When CaCO₃ was combined with asparaginase R, up to 69 % reduction was achieved (CaCO_3_ 100 μmol + R). CaCl_2_ also improved acrylamide reduction when combined with asparaginase R, with the highest reduction reaching up to 65 % (CaCl_2_ + R) in cookies. The combined use of calcium salts and asparaginase resulted in a greater acrylamide reduction than calcium salts alone, highlighting an additive effect. Calcium salts likely modulate the baking environment, inhibiting acrylamide formation, while asparaginase directly reduces the precursor asparagine. Combining CaCl_2_ or ellagic acid with asparaginase R gave lower reductions of acrylamide in crispbread compared to cookies. This could be due to crispbread's lower water activity than cookies. Asparaginase requires sufficient moisture to function effectively, as it catalyzes the hydrolysis of asparagine to aspartic acid (Hendriksen et al., 2009). The drier environment in crispbread limits the enzyme's activity, resulting in a less efficient reduction of acrylamide precursors. Further, the higher baking temperature for crispbread promotes acrylamide formation after asparaginase activity has ceased. The free asparagine content of the wholemeal wheat and rye flour used in this study was 290 mg/kg and 700 mg/kg, respectively, as already reported in our previous study (Musa et al., 2024b). Rye flour generally contains more free asparagine than wheat flour (summarized in Food Drink Europe, 2019), leading to a higher potential to form acrylamide caused by the higher content of the main precursor. Furthermore, the precursor and the more intense thermal treatment applied during baking influence the higher acrylamide content in rye crispbread than wheat cookies.

Both phenolic acids, gallic acid and ellagic acid, respectively, achieved significant reductions of acrylamide in cookies. Ellagic acid alone (100 μmol) reduced acrylamide by 50 % (Fig. 1). Oral et al. (2014) showed that plant extracts and polyphenols can inhibit acrylamide formation. Ellagic acid reduced acrylamide by 69 % in the asparagine-fructose model system and 19 % in biscuits. In the present study, ellagic acid further decreased acrylamide when combined with asparaginase R, with a reduction of up to 89 %. Gallic acid alone caused a reduction of up to 33 %; combined with asparaginase R, it was 65 %. A study where a mix of different phenolic compounds, including gallic acid, was used reported a reduction of acrylamide in fried potato chips by 98 % (Ismial, Ali, Askar, & Samy, 2013). Another study combined aqueous rosemary extract with 40 mg of gallic acid in wheat buns (Hedegaard, Granby, Frandsen, Thygesen, & Skibsted, 2008). They reported a significant decrease in acrylamide using the aqueous extract; however, increasing the amount from 1 % to 10 % of the aqueous extract did not further decrease acrylamide. While in our study a combination of rosemary extract with gallic acid was not investigated, gallic acid and rosemary extract alone provided significant acrylamide reductions of up to 33 % and 50 %, respectively, for cookies. The active components in rosemary extract include carnosic acid, carnosol, and rosmarinic acid. Some phenolic compounds serve as carbonyl-trapping agents that inhibit acrylamide formation during the Maillard reaction (Cheng et al., 2009). According to Constantinou and Koutsidis (2016), in Maillard reaction systems, phenolic antioxidants may react with carbonyl compounds to create adducts via electrophilic aromatic substitution reactions and limit acrylamide formation. Miśkiewicz et al. (2018) also reported increased acrylamide reduction with increasing amounts from 0.1 % to 0.5 % of rosemary extract in shortcrust cookies. Differences in extract efficacy were obtained when shortcrust cookies were baked with or without humid air, and 90 % humidity improved the reductions compared to dry air. While rosemary extract demonstrated an improved reduction of acrylamide levels with increased amounts in both cookies and crispbread in our study, the reductions in crispbread were relatively low and statistically insignificant. Therefore, higher amounts of rosemary extract are recommended specifically for crispbread to enhance its effectiveness. A more detailed investigation of the phenolic compounds present in the rosemary extract will be needed to further clarify its acrylamide-mitigating potential, as some phenolic compounds are known to inhibit acrylamide formation, while others may even promote it. Furthermore, exploring the combination of rosemary extract with ellagic acid or gallic acid offers a promising avenue for future research to identify the most effective strategy for mitigating acrylamide in crispbread. Aside from the regulatory requirements, consumer expectations are important to balance product safety and quality. Therefore, texture, color, and overall sensory properties were evaluated. The texture evaluation showed some significant changes in the hardness of cookies, mainly for the treatment with CaCl_2,_ CaCO_3_, or gallic acid, and fracturability was affected by selected treatments (Fig. 3). Adding salt was reported to create a stronger gluten network, which increases dough stability, dough extensibility, and dough development time, and also decreases water absorption capacity (McCann & Day, 2013), which can explain some of the changes observed in the texture of cookies. Interestingly, the hardness of cookies was again similar to the control when the affected treatments were combined with asparaginase R. In previous studies, the asparaginases also had little or no impact on the textural properties of cookies (Musa, Becker, Oellig and Scherf, 2024a, Musa, Becker, Oellig and Scherf, 2024b).

Calcium salts and phenolic acids showed minimal effect on the color of cookies. The addition of CaCl_2_ showed an increased L*-value compared to the control, which was statistically significant for the 5 μmol and 50 μmol levels. The increase in L*-value was also observed in a study from Açar et al. (2012). The addition of rosemary extract showed significant changes in L*-value for cookies. Similar results were observed from Miśkiewicz et al. (2018). They showed that the color of shortcrust cookies was significantly affected regardless of rosemary extract amounts. On the other hand, rosemary extract did not significantly affect the color properties of crispbread. This is likely because the naturally dark color of the extract blended well with the darker shade of wholemeal rye flour, which was used as the base for crispbread. As a result, any color changes were less noticeable. The more significant differences observed in cookies compared to crispbread can be attributed to these baked goods' distinct composition, structure, and processing. The cookies made from wholemeal wheat flour had a lighter color and softer texture than crispbread, which was made from inherently darker wholemeal rye flour. Therefore, the changes in hardness, fracturability, and color were more noticeable in cookies compared to crispbread, which also had a denser texture, which can hide the changes in color and texture caused by the same components. Although significant changes in texture and color were detected using a texture analyzer and CCell for selected treatments, sensory evaluations showed little or no impact on the texture or color of the chosen treatments. CaCl_2_ treatment (Fig. 4) showed the best overall acceptability compared to the other samples. A study showed that adding CaCl_2_ at 0.1 M left a bitter taste in potato crisps (Mestdagh et al., 2008); however, this amount is significantly higher than our study which might be why no off-taste was detected. They also observed a more intense taste when CaCl_2_ was applied, similar to our research. Increased acceptability of cookies with CaCO_3_ was also reported by Liang et al. (2014). In contrast, Ismial et al. (2013) reported a slight decrease in overall acceptability when CaCl_2_ was applied, while adding magnesium chloride in potato chips showed increased taste and overall acceptability. In another study, lower amounts (0.1 % and 0.5 %) of calcium derivatives, including calcium salts of lactic acid and CaCl_2_, had no noticeable effect on sensory attributes like saltiness, sweetness, or bitterness in cookies (Açar et al., 2012). However, significant changes were observed when the amount was increased to 1.0 %. This highlights that while different salt amounts can vary in their effectiveness at reducing acrylamide, their potential impact on texture, color, and overall acceptability must also be carefully considered. Ellagic acid and rosemary extract (Fig. 4) showed no significant differences in cookies compared to the control sample. Miśkiewicz et al. (2018) also showed that adding rosemary extract in shortcrust cookies at amounts of 0.1 % and 0.2 % baked in dry air did not impact the sensory attributes, while the amount of 0.5 % significantly decreased the sensory properties. Even an 0.2 % amount decreased the sensory assessment for cookies baked in humid air. Since higher amounts than 0.1 % were not assessed using sensory analysis in the current study, further research is needed.

## 5 Conclusion

This study provides novel insights into combining compounds with asparaginase to reduce acrylamide in cookies and crispbread. Adding ellagic acid without asparaginase R (50 mg/kg of Acrylaway L) achieved the highest acrylamide reduction (50 %) in cookies. At the same time, all compounds showed an improved effectiveness when combined with asparaginase R with a decrease of up to 89 %. This approach shows promise as it requires less enzyme than enzyme-only methods, although further studies are needed to confirm this at larger production scales. Acrylamide reduction varied between cookies and crispbread, possibly due to the differences in moisture content and the amount of asparagine in the flour. Crispbread showed a lower reduction, highlighting the importance of matrix-specific strategies. Further research is necessary for crispbread and other baked products because their unique low-moisture matrices and processing conditions limit the effectiveness of existing mitigation strategies. Identifying optimized combinations or novel approaches can help achieve significant acrylamide reductions without compromising product quality. Most treatments had a minimal effect on color, texture, and sensory properties. While calcium salts influenced textural properties, sensory evaluations maintained product acceptability. Further optimization of combinations, including higher amounts and different matrices, is necessary to balance acrylamide reduction with regulatory requirements and quality maintenance across diverse baked products. A key limitation of this study is the lack of sensory evaluation for the crispbread samples. Future research should address this by assessing consumer-relevant attributes like taste and texture across all product types to ensure practical applicability of the mitigation strategies.

## CRediT authorship contribution statement

**Shpresa Musa:** Writing – original draft, Visualization, Methodology, Investigation, Formal analysis, Data curation, Conceptualization. **Claudia Oellig:** Writing – review & editing, Supervision, Resources, Project administration, Conceptualization. **Katharina Anne Scherf:** Writing – review & editing, Supervision, Resources, Project administration, Funding acquisition, Conceptualization.

## Compliance with ethical standards

**Conflict of interest**. The authors declare no competing financial interest.

**Compliance with ethics requirements**. Cookies are typically consumed foods and their sensory evaluation does not require ethical permission. Appropriate protocols for protecting the rights and privacy of all participants in the sensory analysis were utilized during the execution of the research. The publication of all sensory research data was subject to the consent of all participants.

**Informed consent.** All participants in the sensory analysis provided their informed consent to participate in the sensory evaluations and use their information.

## Funding

This IGF project of the FEI was supported within the programme for promoting the Industrial Collective Research (IGF) of the Federal Ministry of Economic Affairs and Climate Action (BMWK), based on a resolution of the German Parliament. Project 01IF21798N. This project was further supported by the Committee on Cereal Research of the Arbeitsgemeinschaft Getreideforschung e.V. (AGF), Detmold. We also thank the German Academic Exchange Service (Deutscher Akademischer Austauschdienst, DAAD) for providing a scholarship to Shpresa Musa. This work is related to COST Action ACRYRED, CA21149, supported by COST (European Cooperation in Science and Technology).

## Declaration of competing interest

The authors declare that they have no known competing financial interests or personal relationships that could have appeared to influence the work reported in this paper.

## Data Availability

Data will be made available on request.

## Appendix A Supplementary data

Supplementary material: Hardness (N) and fracturability (mm) of wholemeal wheat cookies (Table S1) and of wholemeal rye crispbread (Table S2) depending on the different treatments. Image 1
