# Supplementary material for: Mitigation of acrylamide in cookies and crispbread using calcium salts and phenolic acids in combination with asparaginase as well as rosemary extract
Source: Food Chem X. 2025 May 30;28:102605. doi: 10.1016/j.fochx.2025.102605 (PMC12167442; doi:10.1016/j.fochx.2025.102605)
Supplement: Supplementary file 1 — Supplementary material: Hardness (N) and fracturability (mm) of wholemeal wheat cookies (Table S1) and of wholemeal rye crispbread (Table S2) depending on the different treatments. [file mmc1.pdf]

SUPPLEMENTARY MATERIAL

Mitigation of Acrylamide in Cookies and Crispbread Using Calcium Salts and Phenolic Acids in Combination with Asparaginase as well as Rosemary Extract

Shpresa MUSA[1,4], Claudia OELLIG[2], Katharina Anne SCHERF[1,3,4]*

[1] Department of Bioactive and Functional Food Chemistry, Institute of Applied Biosciences, Karlsruhe Institute of Technology (KIT), Karlsruhe, 76131, Germany

[2] Department of Food Chemistry and Analytical Chemistry, Institute of Food Chemistry, University of Hohenheim, Stuttgart, 70599, Germany

[3] Leibniz Institute for Food Systems Biology at the Technical University of Munich, Freising, 85354, Germany

[4] Technical University of Munich, TUM School of Life Sciences, Professorship of Food Biopolymer Systems, Freising, 85354, Germany

Correspondence: Katharina A. Scherf, Leibniz Institute for Food Systems Biology at the Technical University of Munich, Lise-Meitner-Str. 34, Freising, Germany, Email: k.scherf.leibniz-lsb@tum.de

ORCID for Shpresa Musa: 0009-0001-6847-1920

ORCID for Claudia Oellig: 0009-0003-7198-4369

ORCID for Katharina A. Scherf: 0000-0001-8315-5400

Table S1. Hardness (N) and fracturability (mm) of wholemeal wheat cookies with the addition of calcium chloride, calcium carbonate, gallic acid, ellagic acid, and rosemary extract. Abbreviations as follows: $CaCl_2$ (calcium chloride), $CaCO_3$ (calcium carbonate), GA (gallic acid), EA (ellagic acid), RE (rosemary extract), R (50 mg/kg of asparaginase Acrylaway L).

Sample ID	Hardness (N)	Fracturability (mm)
CaCl2_100 umol_1	59.85	1.61
CaCl2_100 umol_2	56.87	2.82
CaCl2_100 umol_3	46.19	2.10
CaCl2_100 umol_4	58.43	2.55
CaCl2_100 umol_5	58.23	1.26
CaCl2_100 umol_6	44.64	1.12
CaCl2_50 umol_1	52.28	1.60
CaCl2_50 umol_2	62.00	2.21
CaCl2_50 umol_3	63.58	1.60
CaCl2_50 umol_4	59.70	1.60
CaCl2_50 umol_5	58.47	1.41
CaCl2_50 umol_6	61.38	1.44
CaCl2_5 umol_1	42.54	2.15
CaCl2_5 umol_2	39.83	3.20
CaCl2_5 umol_3	43.67	1.97
CaCl2_5 umol_4	56.48	2.93
CaCl2_5 umol_5	59.36	1.37
CaCl2_5 umol_6	59.30	1.68
CaCl2_100 umol_R_1	59.62	4.11
CaCl2_100 umol_R_2	59.38	4.63
CaCl2_100 umol_R_3	44.64	3.47
CaCl2_100 umol_R_4	61.32	2.85
CaCl2_100 umol_R_5	53.30	2.50
CaCl2_100 umol_R_6	63.06	3.25
CaCl2_50 umol_R_1	50.54	4.17
CaCl2_50 umol_R_2	51.95	5.65
CaCl2_50 umol_R_3	46.33	5.90
CaCl2_50 umol_R_4	50.83	4.67
CaCl2_50 umol_R_5	50.45	5.01
CaCl2_50 umol_R_6	36.62	5.21
CaCl2_5 umol_R_1	54.76	1.43
CaCl2_5 umol_R_2	58.39	1.30
CaCl2_5 umol_R_3	58.65	1.43
CaCl2_5 umol_R_4	57.54	1.16
CaCl2_5 umol_R_5	60.53	2.09
CaCl2_5 umol_R_6	57.37	1.22
CaCO3_100 umol_1	9.83	11.82
CaCO3_100 umol_2	36.24	4.67
CaCO3_100 umol_3	40.59	2.66
CaCO3_100 umol_4	38.70	2.91

CaCO3_100 umol_5	35.85	2.04
CaCO3_100 umol_6	45.68	1.86
CaCO3_50 umol_1	25.71	8.01
CaCO3_50 umol_2	36.73	3.76
CaCO3_50 umol_3	36.80	2.46
CaCO3_50 umol_4	20.64	6.14
CaCO3_50 umol_5	26.47	10.22
CaCO3_50 umol_6	36.25	4.85
CaCO3_5 umol_1	44.50	4.51
CaCO3_5 umol_2	45.89	4.01
CaCO3_5 umol_3	48.12	11.37
CaCO3_5 umol_4	49.27	3.08
CaCO3_5 umol_5	49.16	4.69
CaCO3_5 umol_6	41.56	6.01
CaCO3_100 umol_R_1	61.53	2.09
CaCO3_100 umol_R_2	57.63	1.18
CaCO3_100 umol_R_3	57.11	1.42
CaCO3_100 umol_R_4	51.41	1.38
CaCO3_100 umol_R_5	53.75	2.20
CaCO3_100 umol_R_6	58.90	1.42
CaCO3_50 umol_R_1	61.09	2.00
CaCO3_50 umol_R_2	44.73	1.98
CaCO3_50 umol_R_3	47.39	2.84
CaCO3_50 umol_R_4	49.46	3.46
CaCO3_50 umol_R_5	49.73	2.21
CaCO3_50 umol_R_6	46.73	3.47
CaCO3_5 umol_R_1	50.63	2.35
CaCO3_5 umol_R_2	42.29	1.70
CaCO3_5 umol_R_3	43.09	1.65
CaCO3_5 umol_R_4	54.29	2.18
CaCO3_5 umol_R_5	57.16	3.29
CaCO3_5 umol_R_6	37.95	3.37
GE_100 umol_1	23.84	2.67
GE_100 umol_2	23.01	3.83
GE_100 umol_3	11.70	2.43
GE_100 umol_4	25.87	3.16
GE_100 umol_5	27.55	3.96
GE_100 umol_6	21.22	2.24
GE_50 umol_1	37.12	2.71
GE_50 umol_2	33.26	3.30
GE_50 umol_3	38.09	3.77
GE_50 umol_4	40.37	2.03
GE_50 umol_5	50.01	1.75
GE_50 umol_6	25.52	2.78
GE_5 umol_1	33.45	4.16
GE_5 umol_2	29.34	5.99
GE_5 umol_3	20.24	4.32
GE_5 umol_4	23.54	5.99

GE_5 umol_5	20.54	5.90
GE_5 umol_6	21.97	4.12
GE_100 umol_R_1	58.77	1.32
GE_100 umol_R_2	63.36	1.41
GE_100 umol_R_3	63.40	2.03
GE_100 umol_R_4	57.49	1.22
GE_100 umol_R_5	54.05	1.24
GE_100 umol_R_6	54.04	1.66
GE_50 umol_R_1	58.39	1.51
GE_50 umol_R_2	50.95	3.40
GE_50 umol_R_3	55.96	2.17
GE_50 umol_R_4	61.87	3.00
GE_50 umol_R_5	52.05	2.20
GE_50 umol_R_6	61.04	1.63
GE_5 umol_R_1	38.46	4.62
GE_5 umol_R_2	42.32	6.16
GE_5 umol_R_3	34.05	4.62
GE_5 umol_R_4	38.13	4.71
GE_5 umol_R_5	22.75	4.35
GE_5 umol_R_6	34.73	4.95
EA_100 umol_1	59.16	2.20
EA_100 umol_2	56.81	3.01
EA_100 umol_3	50.11	4.43
EA_100 umol_4	58.13	1.50
EA_100 umol_5	58.70	5.20
EA_100 umol_6	42.52	1.91
EA_50 umol_1	38.77	5.17
EA_50 umol_2	48.89	3.06
EA_50 umol_3	47.99	3.11
EA_50 umol_4	57.24	1.97
EA_50 umol_5	61.40	4.54
EA_50 umol_6	49.73	1.81
EA_5 umol_1	48.87	2.91
EA_5 umol_2	54.39	2.04
EA_5 umol_3	38.98	2.50
EA_5 umol_4	38.64	2.07
EA_5 umol_5	47.52	2.00
EA_5 umol_6	40.17	3.81
EA_100 umol_R_1	57.66	1.29
EA_100 umol_R_2	61.05	2.70
EA_100 umol_R_3	61.76	2.13
EA_100 umol_R_4	56.09	1.91
EA_100 umol_R_5	57.61	2.50
EA_100 umol_R_6	56.65	2.41
EA_50 umol_R_1	47.72	2.15
EA_50 umol_R_2	56.33	1.45
EA_50 umol_R_3	61.23	3.26
EA_50 umol_R_4	55.77	2.54

EA_50 umol_R_5	55.81	1.99
EA_50 umol_R_6	63.18	1.77
EA_5 umol_R_1	47.41	2.28
EA_5 umol_R_2	47.62	3.99
EA_5 umol_R_3	47.12	10.26
EA_5 umol_R_4	61.03	1.74
EA_5 umol_R_5	62.64	2.13
EA_5 umol_R_6	63.44	2.55
RE_0.01%_1	49.74	2.26
RE_0.01%_2	44.97	2.27
RE_0.01%_3	51.14	2.00
RE_0.01%_4	31.51	1.86
RE_0.01%_5	39.46	1.98
RE_0.01%_6	43.92	1.18
RE_0.1%_1	55.14	3.68
RE_0.1%_2	34.31	5.62
RE_0.1%_3	57.30	2.98
RE_0.1%_4	45.93	3.92
RE_0.1%_5	44.25	11.26
RE_0.1%_6	56.16	4.74
RE_0.2%_1	36.63	1.16
RE_0.2%_2	47.67	2.21
RE_0.2%_3	37.91	1.23
RE_0.2%_4	49.51	1.27
RE_0.2%_5	43.58	1.32
RE_0.2%_6	49.97	1.49
Control_1	50.08	1.83
Control_2	53.33	1.56
Control_3	55.53	1.30
Control_4	49.59	1.05
Control_5	50.04	2.93
Control_6	55.45	1.55
Control_R_1	59.23	1.38
Control_R_2	38.54	2.01
Control_R_3	51.50	2.42
Control_R_4	58.43	1.13
Control_R_5	55.21	1.99
Control_R_6	60.43	1.38

Values in grey were identified as outliers.

Table S2. Hardness (N) and fracturability (mm) of wholemeal rye crispbread with the addition of calcium chloride, ellagic acid, and rosemary extract. Abbreviations as follows: $CaCl_2$ (calcium chloride), EA (ellagic acid), RE (rosemary extract), R (50 mg/kg of asparaginase Acrylaway L).

Sample ID	Hardness (N)	Fracturability (mm)
CaCl2_100 umol_R_1	53.42	1.56
CaCl2_100 umol_R_2	55.87	2.11
CaCl2_100 umol_R_3	53.48	2.20
CaCl2_100 umol_R_4	53.06	1.94
CaCl2_100 umol_R_5	58.73	2.09
CaCl2_100 umol_R_6	57.15	2.12
CaCl2_50 umol_R_1	55.72	2.30
CaCl2_50 umol_R_2	54.28	1.55
CaCl2_50 umol_R_3	56.53	1.91
CaCl2_50 umol_R_4	58.11	2.82
CaCl2_50 umol_R_5	52.60	1.99
CaCl2_50 umol_R_6	51.56	1.80
CaCl2_5 umol_R_1	58.58	2.34
CaCl2_5 umol_R_2	58.22	1.54
CaCl2_5 umol_R_3	54.83	2.13
CaCl2_5 umol_R_4	54.46	2.54
CaCl2_5 umol_R_5	55.33	1.23
CaCl2_5 umol_R_6	56.25	1.72
EA_100 umol_R_1	53.76	1.56
EA_100 umol_R_2	52.72	1.50
EA_100 umol_R_3	53.60	2.02
EA_100 umol_R_4	52.80	1.92
EA_100 umol_R_5	52.62	2.20
EA_100 umol_R_6	53.81	1.87
EA_50 umol_R_1	50.75	2.29
EA_50 umol_R_2	49.37	1.81
EA_50 umol_R_3	49.13	1.59
EA_50 umol_R_4	48.68	1.48
EA_50 umol_R_5	50.59	1.45
EA_50 umol_R_6	47.53	1.46
EA_5 umol_R_1	51.89	2.16
EA_5 umol_R_2	48.32	1.57
EA_5 umol_R_3	52.68	1.86
EA_5 umol_R_4	51.34	2.32
EA_5 umol_R_5	47.50	1.69
EA_5 umol_R_6	51.23	1.91
RE 0.01%_1	50.68	2.30
RE 0.01%_2	52.38	2.12
RE 0.01%_3	49.43	2.85
RE 0.01%_4	50.44	2.18
RE 0.01%_5	50.59	2.03
RE 0.01%_6	49.71	2.19
RE 0.1 %_1	49.31	2.36

RE 0.1 %_2	50.51	2.54
RE 0.1 %_3	50.74	2.77
RE 0.1 %_4	51.77	2.15
RE 0.1 %_5	52.28	2.61
RE 0.1 %_6	49.74	2.08
RE 0.2%_1	49.35	2.75
RE 0.2%_2	50.82	2.21
RE 0.2%_3	49.15	2.65
RE 0.2%_4	49.26	2.89
RE 0.2%_5	50.83	2.46
RE 0.2%_6	52.60	2.17
Control_1	51.83	2.12
Control_2	52.95	1.88
Control_3	53.33	2.47
Control_4	50.42	2.14
Control_5	53.93	2.36
Control_6	52.52	1.78
